# The Plethora of RNA–Protein Interactions Model a Basis for RNA Therapies

**DOI:** 10.3390/genes16010048

**Published:** 2025-01-02

**Authors:** Stephen J. Dansereau, Hua Cui, Ricky P. Dartawan, Jia Sheng

**Affiliations:** Department of Chemistry, The RNA Institute, University at Albany, SUNY, 1400 Washington Ave Extension, Albany, NY 12222, USA; sdansereau@albany.edu (S.J.D.); hc875@cornell.edu (H.C.);

**Keywords:** RNA, RNA World, ribozyme, riboswitch, ribosome, spliceosome, nucleotide-repeats, aptamers, therapeutic

## Abstract

The notion of RNA-based therapeutics has gained wide attractions in both academic and commercial institutions. RNA is a polymer of nucleic acids that has been proven to be impressively versatile, dating to its hypothesized RNA World origins, evidenced by its enzymatic roles in facilitating DNA replication, mRNA decay, and protein synthesis. This is underscored through the activities of riboswitches, spliceosomes, ribosomes, and telomerases. Given its broad range of interactions within the cell, RNA can be targeted by a therapeutic or modified as a pharmacologic scaffold for diseases such as nucleotide repeat disorders, infectious diseases, and cancer. RNA therapeutic techniques that have been researched include, but are not limited to, CRISPR/Cas gene editing, anti-sense oligonucleotides (ASOs), siRNA, small molecule treatments, and RNA aptamers. The knowledge gleaned from studying RNA-centric mechanisms will inevitably improve the design of RNA-based therapeutics. Building on this understanding, we explore the physiological diversity of RNA functions, examine specific dysfunctions, such as splicing errors and viral interactions, and discuss their therapeutic implications.

## 1. Part I: RNA–Protein Evolution and Biology

Darwin’s Origin of Species postulated that all life descended from one primordial form. The earliest genetic material is believed to be RNA, which, according to the RNA World Theory, preceded the time of protein synthesis and function as enzymes [1]. Crick postulated that early ribosomes could entirely be made of RNA [2], and polypeptide synthesis without proteins has since been demonstrated as possible, albeit much less efficiently [2,3,4], casting the ribosome itself as a ribozyme. Proteins may facilitate this peptidyl transfer reaction by guiding the ribosome to a fold conducive for catalysis [5].

Still, the notion that RNA evolved independently of peptides remains ambivalent. The purported self-replication ability of ribozymes is yet to be shown experimentally [6], though Synak et al. (2020) reported the possibility of evolutionary selection for such a polymerase [7]. Moreover, Tagami et al. (2023) observed certain varieties of peptides that facilitate RNA synthesis through either their binding to RNA polymerase or formation of the ribozyme’s native core [6]. Such prebiotic peptides could have begun as noncoded amino acid chains from Miller’s primordial soup [8] that were amenable to polyribonucleotides in this prebiotic environment [9].

Amino acid-binding riboswitches may very well have laid the foundation for the deliberate formation of peptides [10], which later became intertwined with cellular signaling networks as the first RNA-binding proteins (RBPs) [11]. Thus, whether RNA and proteins evolved concurrently or sequentially, numerous RNA molecules and structures, including nucleotide-derived coenzymes, self-processing ribozymes, metabolite-binding riboswitches, and ribosomes, play vital roles in gene expression and cellular physiology [12].

### 1.1. Riboswitch Folding and Dynamics

As the name suggests, riboswitches are non-coding RNAs [13] that function as genetic switches [14]. Consisting of an aptamer and expression platform, the former operates as a sensor so as to cue its downstream component in response to the intracellular environment [15]. Such tuning of gene expression [16], per small metabolite, elemental ion, or other effector-types [17], harkens to the RNA World Theory [18]. To this point, riboswitches are capable of binding their target or otherwise adopting their prescribed regulatory structures without proteins, though, again, proteins may support these molecules’ structural versatility [19].

Although some level of conformational selection in the apo state has been observed [20], the dynamics among these regulatory folds are nonetheless crucial [21] for governing mRNA transcription, translation, and decay [22]. Indeed, distinct conformations were observed among the apo and bound states of the S-adenosylmethionine (SAM)-responsive riboswitches [23]. General conformational reorganizations involve loop–loop interactions and coaxial stacking while retaining intrinsically disordered regions for effector accessibility to the binding site [18].

Superseding the established two-state “on-off” system, Reining et al. (2013) investigated the adenine sensing riboswitch in *Vibrio vulnificus* using ^1^H-^15^N heteronuclear chemical exchange NMR spectroscopy, demonstrating a third conformation inherent to its mechanism [24]. Single-molecule FRET (smFRET) not only corroborated a kissing loop fold of this three-way junctional purine riboswitch aptamer, but further conveyed its persistence through docked and undocked conformations [25]. Thus, in-tandem, NMR and smFRET are powerful tools for comparing folded conformations and measuring their exchange dynamics [26].

Some riboswitches operate via the alternative pairing between nucleotides separated by some intramolecular distance [27], thereby bringing them together and facilitating the removal of an intron [28]. Such a regulation of alternative splicing by riboswitches is common across eukaryotes [29,30], and the knowledge of the binding site and splicing machinery can be exploited to engineer a riboswitch that upregulates a given protein [31].

### 1.2. Spliceosomes and Pre-mRNA Processing

The spliceosome is a ribonucleoprotein (RNP) complex that catalyzes pre-mRNA splicing to help pre-mRNA mature into mRNA by removing introns [32]. Exclusive to eukaryotic genes, the spliceosome’s structure is highly conserved among yeasts and metazoans [33]. Microorganisms, such as *Escherichia coli* or the unicellular eukaryotic *Tetrahymena thermophila*, have self-splicing capabilities [34].

Roughly 200 proteins and five snRNAs constitute the spliceosome, and the recognition of the splice site by multiple RNAs and proteins affords a high accuracy to the splicing process [33]. The two types of spliceosomes, major and minor, splice different exons, but can work together to influence mRNA processing where there are minor introns [35].

**Figure 1:**
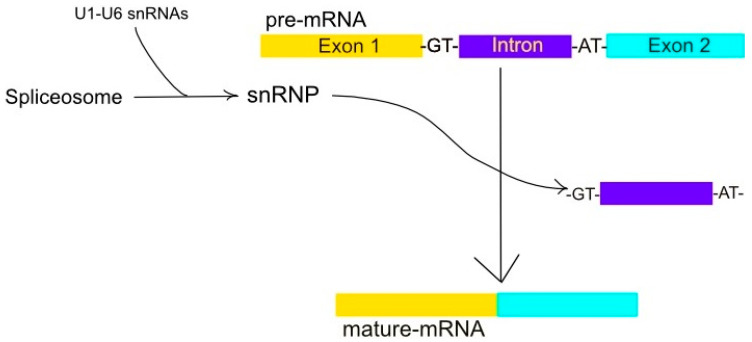
A ribonucleoprotein complex constitutes the spliceosome. The spliceosome, a ribonucleoprotein complex consisting of snRNAs U1-6, is instrumental in converting pre-mRNA into mature-mRNA via the excision of non-coding intron sequences, typically bordered by GT and AT nucleotides, of the mRNA. This allows the expressed regions, or exons, to merge into a single, mature mRNA.

The major spliceosome splices 99.5% of introns with GU at the 5′ splice site and AG at the 3′ splice site [36]. The major spliceosome is composed of the snRNAs predominant in metazoans, plants, and fungi [36,37]. This spliceosome removes introns with GT-AT and occasionally AT-AC and GC-AG boundaries [36], as illustrated in Figure 1. The major spliceosome works by interactions between snRNAs and spliceosome proteins, in addition to their interactions with the pre-RNA substrate [36].

The minor spliceosome only splices 0.5% of introns and specializes in severing atypical U12-type introns [35]. Both the major and minor spliceosomes share an overall common secondary structure and span most eukaryotic lineages [35,36].

#### 1.2.1. Small Nuclear (sn) RNAs and Pre-mRNA Form the Spliceosome

A number of factors interact with pre-mRNA to form the spliceosome and distribute the pre-mRNAs’ reactive groups for catalysis. Of these are various snRNPs and non-snRNPs alike, each of the former consisting of its own snRNAs [37]. The rearrangements among these snRNAs throughout the splicing process imparts structural fluidity among the many RNA–RNA and RNA–protein interactions that constitute a spliceosome [38]. The subsequent and spontaneous dissociation of these molecules from the spliceosome primes it for another round of splicing [39].

#### 1.2.2. Polyadenylation in mRNA Stability and Translation

Adding a poly(A) tail to post-transcriptional mRNA can increase the stability in mRNA in cells [40]. Most poly(A) tails are located on the 3′ untranslated regions (3′ UTRs). The tail is recognized by nuclear export proteins, which help mature mRNA enter the cytoplasm from the nucleus [40]. The tail also binds to poly(A)-binding proteins (PABP), which are involved in translation efficiency, thereby linking the 5′ end to the 3′ end in mRNA and facilitating ribosomal attachment and recruitment [41].

### 1.3. RNA Stability and Decay

Gene expression is dependent on the stability of both coding and non-coding RNAs [42,43], as the former are receptive to environmental stimuli so as to modulate protein production accordingly [44]. Mechanistically, mRNA decay rates are dictated by bound proteins and non-coding RNAs [45] that facilitate mRNA degradation through various pathways [46,47].

A common approach among eukaryotes is the removal of the poly(A) tail in a process known as deadenylation and the subsequent exonucleolytic degradation in either the 5′ or 3′ direction [48]. Funakoshi et al. (2007) have demonstrated the eukaryotic elongation factor (eRF3) to work in concert with PABPC1 and the deadenylation complexes Pan2-Pan3 and Caf1-Ccr4 [49] in a sequence-specific manner [50]. These shortened poly(A) tails are then recognized by the Lsm1-7/Pat1 complex, which signal for the removal of the 5′ cap by the decapping enzyme complex Dcp1p/Dcp2p [51]. Consequently, mRNA degradation in the 5′-3′ direction ensues [52], though Audebert et al. (2024) recently demonstrated an independence between deadenylation and decapping [53].

The presence of AU-rich elements (AREs) in the 3′UTR of unstable mRNAs offers an alternate means of regulation over gene expression [54,55,56], and Park-Lee et al. (2003) demonstrated the efficacy of these regions in recruiting their binding partners of as little as 13 residues [57]. Moreover, two RNA-recognition motif (RRM) domains constituting HuD have been crystalized in complex with a short oligonucleotide fragment of class II AREs [58]. These Hu proteins belong to the ELAV family of RBPs, which comprises four members in humans: HuR occurs is all proliferating cell-types while Hel-N1, HuC, and HuD are confined to terminally differentiated neurons [59].

The overlap or close proximity among 3′ UTR regulatory sequences bound by RBPs and miRNAs suggests a concentration-dependent accessibility and, thus, competition among regulatory processes [60,61].

### 1.4. Telomerase RNA Component (TERC) and Cell Senescence

Telomeres are important for delaying the senescence of cells because every time cells split, the ends of DNA strands are excised [62]. Telomerase replicates telomeres and TERC is involved in synthesizing telomerase on chromosomal ends [63]. Non-coding RNAs are important in delaying senescence. Flores et al. (2001) demonstrated that TERC was restored in mice with short telomere length, increasing chromosomal stability; reviving telomere activity reversed some effects of aging in mice [64]. Moreover, Rudolph et al. (2024) showed that hepatic damage in the liver damages TERC length, resulting in senescence in liver cells [63]. The effects of senescence were shown to be reversible by TERC transfer, indicating the possible therapeutic use of TERC transfer, in the future, to combat liver cirrhosis [64,65]. Therapeutics in the pipeline targeting TERC, such as a possible gene therapy, has been postulated to prevent cancer [65]. For instance, Koscioleck et al. (2003) found that siRNAs targeting TERC or TERT (telomerase reverse transcriptase) in cancer cells attenuate telomerase activity [64,66].

### 1.5. Ribosomes in Translation and Regulation

Ribosomes present with structural dissimilarities, suggesting subspecialties based on their own binding specificities [67]. Such a heterogeneity is conducive to the rapid changes in protein expression, suggesting that ribosomal subpopulations govern the translation of their assigned mRNAs [68]. This has been evidenced in diploid studies using *Saccharomyces cerevisiae*, whereby the depletion of one copy of a ribosomal protein gene compromised the translation of localized ASH1 mRNA [69].

Ribosomal proteins (RPs) are instrumental in DNA repair, cellular development, and cellular differentiation [70]. Given the ribosome’s many enzymatic components, it has been referred to as the ribozyme, whereby ribosomal RNAs (rRNAs) have catalytic activity [3]. What separates eukaryotic rRNAs from their prokaryotic counterparts are their expansion segments and RP-specificity. This enhances their dual role during the translation of decoding genetic information and catalyzing peptide bond formation [71,72].

Small and large ribosomal subunits are synthesized in the nucleolus from the precursor 28S, 18S, and 5.8S rRNAs and their assembly with ribosomal proteins [73]. Following their export to the cytoplasm, these ribosomal subunits are further processed into mature 40S and 60S ribosomal subunits [74].

The activity of various ribosomal proteins has been shown to be dependent upon non-ribosomal proteins. Fuchs et al. (2011) investigated this dependence: By separately purifying actively translating and non-translating ribosomes in HeLa cells, a mass spectrometry analysis revealed glycogen synthase 1 (GYS1) to be specifically associated with polysomes [69]. As a member of the family of glycosyltransferases, this protein catalyzes the rate-limiting step during glycogen biosynthesis [75].

#### Ribosome Biosynthesis and Ribosomopathies

Aside from their role in viral replication, ribosomes are also believed to play a role in the cell’s stress response [76]. Ribosome biogenesis, facilitated by nutrient rich conditions and hindered under cellular stress, is a necessary component in regulating cell growth and proliferation [77]. The disruption of this process is believed to cause ribosomopathies associated with various cancers and metabolic disorders, thus initiating the p53 signaling pathway and, consequently, apoptosis [78].

## 2. Part II: RNA Dysfunction, Disease, and Drug Targets

RNA dysfunction leads to numerous diseases. Some dysfunctions include transcriptional errors where the mRNA formed is faulty due to mutations in the transcription factors, promotors, or enhancers [79]. An example of such a disease is the Fragile X Syndrome [79]. The failure to polyadenylate pre-mRNA could result in oculopharyngeal muscular dystrophy (OCMD), thalassemias, and neurodevelopmental disorders [79]. Alternative splicing dysregulation leads to diseases such as myotonic dystrophy. Viruses and bacteria can exploit RNA-based cellular machinery. However, suitable drug targets and therapeutics can be derived from studying malformed RNA, which will be further elaborated on in Part III of this review.

### 2.1. Dysregulation in Alternative Splicing and Trinucleotide-Repeat Disorders

As already described, the purpose of the spliceosome is to facilitate alternative splicing, which expands the coding repertoire of our genes. In turn, this allows for the environment-specific expression of the various possible isoforms. The ratio of isoforms becomes altered during diseases, including cancers, muscular dystrophies, and neurological diseases [80]. Calibrating the concentration of alternative splicing factors to given isoform ratios is a current area of investigation [80], as in the case of myotonic muscular dystrophy.

#### 2.1.1. CUG-Repeats in DM1

Myotonic dystrophy type 1 (DM1) is the most prevalent form of adult-onset muscular dystrophy but lacks a disease-targeting treatment [81]. Its cause has been attributed to an extended CTG repeat tract in the 3′ UTR of the dystrophia myotonica protein kinase (DMPK) gene [82]. Consequently, a cytotoxic CUG-repeat RNA is transcribed and retained in the nucleus [83], disrupting the function of RBPs of the muscleblind-like (MBNL) and CUG-BP Elav-like (CELF) families [71,84].

**Figure 2:**
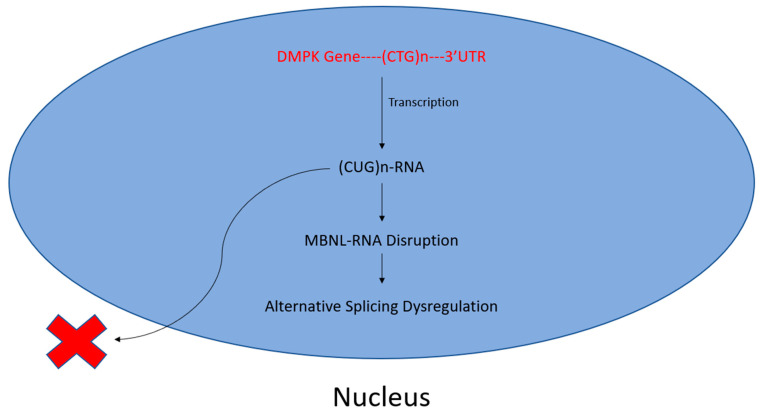
Toxic CUG-repeats disrupt MBNL–RNA interactions. A buildup of CTG repeats in the 3′ UTR of the DMPK gene leads to increased CUG–RNA interactions following transcription. Subsequently, otherwise necessary, MBNL–RNA interactions are disrupted, perturbing alternative splicing. This manifests as myotonic muscular dystrophy.

The subsequent sequestration of MBNL proteins and upregulation of CELF proteins perturb alternative splicing, translation, polyadenylation, microRNA processing, and mRNA localization. Such a dysregulation of alternative splicing espouses variable disease symptoms, including the mis-splicing of muscle-specific chloride channel (CLCN1), cardiac troponin T (TNNT2) and insulin receptor (IR) mRNAs, resulting in myotonia, cardiac defects, and insulin resistance, respectively [71,72]. A schematic of this CTG repeat-mediated disruption of RNA–protein interactions is shown in Figure 2.

Berglund et al. (2016) linked levels of RNA-binding protein MBNL1 to its role in splicing events crucial for muscle, cardiovascular, and nervous system development [80]. Imbalances in MBNL1-RNA complexes, along with interactions with MBNL2 and MBNL3, can result in myotonic dystrophy. Specifically, MBNL proteins binding to the YGCY clusters of toxic RNA leads to both myotonic dystrophy types 1 and 2. Moreover, altering the YGCY clusters of the intron splicing silencer (ISS) immediately upstream allowed for the MBNL dose-dependent splicing activity to be analyzed [80]. By studying 44 DM1 patients and 11 healthy controls, it was discovered that the variability in splicing events between DM1 patients could be biomarkers for disease severity [80].

Some small molecule inhibitors of the MBNL–CUG interaction have been developed to modulate the production and stability of CUG repeats. Alternate drug targets that modulate these levels are kinases [71]. For example, the anti-sense oligonucleotide Balisforsen decreases levels of cytotoxic RNA repeats by targeting DMPK [85].

Exploiting the multistep pathogenic mechanism of DM1, Berglund et al. sought to combine the antibiotic erythromycin and the trypanicide furamidine as a multifaceted DM1 treatment [71]. Both small molecules have previously been shown to reverse mis-splicing in DM1 mouse models and are believed to work by displacing and thereby freeing MBNL from its complex with CUG RNAs. A combination treatment of each, in a transgenic mouse expressing the human skeletal actin (HSA) with 220 CUG repeats, demonstrated additive effects three times greater than either stand-alone treatment [71].

#### 2.1.2. Microsatellite Aggregates Form Stable, Cytotoxic RNA Foci

The aforementioned short-tandem repeats are microsatellites and constitute 2–12 base pair stretches of DNA. Found in both coding and non-coding regions, these tracts are intrinsically unstable and display high variability in gene site, length, and function. The latter is not limited to regulatory roles, DNA repair, and non-canonical translation. However, when such repeats occur in the thousands, either loss- or gain-of-function mutations occur with ensuing pathology [86].

Most diseases of this etiology are inherited in a dominant fashion, and the accumulation of expanded-repeat RNAs within the cell forms complexes called RNA foci [87]. These foci gain their stability from both Watson–Crick and non-canonical base pairing, forming secondary structures such as imperfect stem-loops [88]. These include single A-form double helices for shorter repeats that accommodate U–U mismatches, while longer repeat RNAs comprise an ensemble of stem–loop structures [86,87,88].

RNA foci were first identified in DM1-diseased fibroblasts and myofibrils, which contain nuclear clumps of dystrophia myotonica protein kinase (DMPK) mRNA with the expanded CUG repeats [89]. Although RNA normally supports RNP phase separations for regulatory purposes, repeat expansions exaggerate this transition with cytotoxic consequences [34,86].

#### 2.1.3. CCTG-Repeats and DM2

Myotonic dystrophy II (DM2) is a disorder caused by an RNA gain-of-function: An unstable CCTG repeat expansion on intron 1 of the CNBP gene in chromosome 3q21 results in the formation of hairpin structures [90]. The disorder is, in most cases, less debilitating than DM1 and lacks a clear common presentation at birth [91]. The CNBP gene codes for a zinc-finger RNA binding protein that mediates transcriptional repression [91,92].

DM2 presents possible RNA drug targets because the disorder involves the inability of MBNL1 to participate in alternative splicing [93]. Therefore, therapeutics involving small molecule treatment and anti-sense oligonucleotides are worth investigating because of their potential ability to disrupt the sequestration of MBNL1.

#### 2.1.4. CGG-Repeats and FXTAS

Fragile X Syndrome (FXTAS) is another RNA gain-of-function disorder caused by CGG repeat expansions in the FMR1 gene on the X chromosome [94]. When the amount of CGG repeats exceed 200, the ensuing hypermethylation leads to the transcriptional silencing of the FMRP gene promoter and a consequent deficiency in this protein [95]. The inherited disorder is known to be the most prevalent cause of mild-to-severe intellectual disabilities and has high variance in presentation among such patients [94,95]. This hallmark of FXTAS manifests because the FMRP protein is normally tasked with removing interfering proteins around synapses. Thus, scant FMRP greatly diminishes synaptic plasticity [96].

#### 2.1.5. CTG*CAG-Repeats and SCA8

SCA8 is a disease involving slowly intensifying ataxia that occurs when the two overlapping genes, ATXN8OS/ATXN8, include abnormal (CTG*CAG)n repeat expansions identified through a proband, a type of molecular genetic testing [97,98]. Like the previous disorders discussed in this section, SCA8 expansion occurs in the non-coding region, resulting in toxic RNA that can disrupt normal RNA functions by sequestering functional RNA [99]. One result of overexpression in the SCA8 mutation is the production of polyglutamine proteins and CUG/CAG transcripts [100]. This leads to the sequestration of the MBNL1 gene, thereby creating motor deficits [97,101].

### 2.2. Viruses

Viruses infect hosts by evading their immune response and hijacking replication machinery, namely, ribosomes and translation factors, in order to synthesize viral proteins [102].

An invading virus can have a genome comprising either DNA or RNA surrounded by a protein shell forming a capsid [103]. Normally, cellular mRNAs are transcribed in the nucleus, facilitated at their 5′ end by a 7-methyl-guanosine (m^7^G) cap binding to the cap-binding protein eIF4E (eukaryotic translation initiation factor 4E) [103]. Translation can occur cap-dependently or cap-independently, and the latter is often employed by viral mRNAs [76].

#### 2.2.1. Cap-Independent Translation Targets Ribosome

Some viruses have their own RNA structural elements upstream of the open reading frame. Such internal ribosomal entry sites (IRESs) operate independent of the 5′ methyl cap and are, thus, referred to as cap-independent translation [104]. This direct means of targeting ribosomes and translation factors alters both their availability and associated posttranslational modifications [105]. The latter was made possible by the viral IRES’s affinity to certain ribosomal proteins such as RACK1 [105].

**Figure 3:**
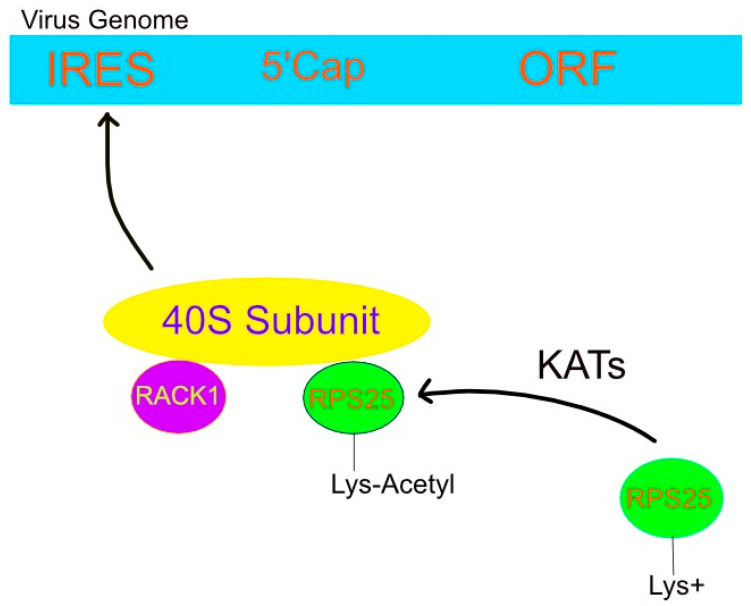
Viral IRES attracts 40S subunit via lysine acetylation of RPS25. Lysine acetyltransferases neutralize the positive charge on lysines, thereby allowing RPS25, along with RACK1, to bind the 40S ribosomal subunit. This complex facilitates the so-called cap-independent translation of the viral genome by freeing it of eukaryotic transcription factors that target the 5′ cap. Instead, the small ribosomal complex interacts with the virus’ IRES and, consequently, translates its genome using host machinery.

RACK1 is a 40S ribosomal subunit protein required for the replication of certain viruses [105]. Its function during IRES-mediated translation appears distinct from its eS25 counterpart, as illustrated by the inability of ribosomes lacking eS25 to bind to the IRES and the consequent translational inhibition [106]. However, a loss of RACK1 did not have the same effect, and the wide array of RACK1 functions may impart both proviral and antiviral outcomes [105]. The dependency of ribosomal recruitment to the IRES is illustrated in Figure 3.

Specifically, Cryo-EM places RPS25 on the 40S subunit near mRNA entry, and Fuchs et al. (2015) used fluorescent labeling to study the RPS25 recruitment to an HCV IRES [107]. Introduction of their RPS25-SNAP fusion protein within RPS25 KO cells restored translation. Moreover, dynamic FRET revealed flexibility in this protein and its interaction with the HCV IRES [107].

#### 2.2.2. Viruses Target Post-Translational Modifications

Post-translational modifications encompass nature’s repertoire of 20 amino acids, primarily eliciting methylation, phosphorylation, and acetylation at the amino acid chain’s N- or C-termini. These modifications to host cell proteins and factors can be imparted by viral enzymes, thereby disrupting protein folding and cell signaling. For instance, the acetylation of eL24 at K27 was found to decrease the association of the modified eL24 with polysomes [105]. Moreover, a comparison of 31 ribosomal proteins by Yu et al. (2005) revealed inconsistent modifications between IRES-bound and native 40S ribosomal subunits [106,107].

Viruses exploit host post-translational modifications by various methods including ubiquitination and less common approaches, such as ISGylation, SUMOylation, and ADP ribosylation [108]. The occurrences of these processes attenuating and promoting viral infections have been reported. An example of the former is the ubiquitination of Dengue (DENV) NS3, which tags the virus for proteolytic degradation [109]. The latter involves ubiquitination, as seen with the Ebola virus protein VP35; the indiscriminate ubiquitination of the viral polymerase subunit inadvertently enhances its enzymatic activity [110].

The cap-independent method employs the virus’ IRES within the 5′ untranslated region of their genome. However, the mechanistic details of this IRES-mediated translation are unknown, something compounded by the evolution of IRES across different viruses. One commonality shared among viruses is their need for the ribosomal protein eS25 (RPS25) in order to initiate translation [106,111].

#### 2.2.3. tRNA Modifications in Antibiotic Design

Understanding the mechanisms of translational modifications offers a means of antibiotic design [112,113]. One site of particular interest along the codon–anticodon complex is position-34, also known as the wobble position [114,115]. As the third base pair along the three-letter codon, the stability of the first two base pairs affords some flexibility [116], thereby tolerating non-canonical base pairs [117] essential for advancing the ribosome properly along the reading frame [118]. The absence of these modifications in humans can induce frameshift mutations [119], but disrupting bacterial translation machinery provides an antimicrobial approach. Bacterial tRNA-2-selenouridine synthase (SelU) is a drug target responsible for derivatizing 2-uridine of this anticodon position with either a geranyl group or selenium [118].

#### 2.2.4. SelU-Mediated Geranylation and Selenation of tRNA Affects Translational Fidelity

**Figure 4:**
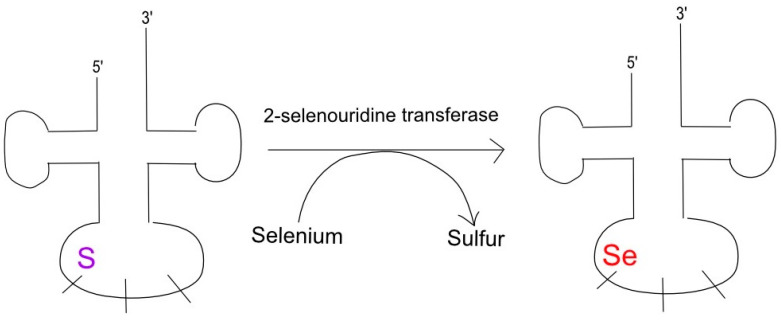
SelU causes selenation at tRNA’s 5′ wobble position. Modifications of tRNA nucleosides, especially around the wobble and anticodon positions, can induce mutations. In particular, 2-selenouridine transferase catalyzes the conversion of sulfur to selenium, leading to a frameshift mutation and the associated pathology.

These two modifications are centered around the role of selenium, namely, that of 2-selenouridine in tRNA anticodons [120]. Most seleno-tRNAs code for lysine, glutamate, and glutamine with their isoacceptors containing 5-methylaminomethyl 2-selenouridine in the wobble position of their anticodons [120]. This process begins as the original tRNA transcript’s uridine is modified with sulfur and yields 2-selenouridine upon the substitution of sulfur with selenium, as illustrated in Figure 4 [121].

Upon treating the RNA isolated from bacteria with nuclease P1, Dumelin et al. (2012) found eight species that contained the modified nucleobases mnm5s2U and cmnm5s2U [122]. Geranyl modifications, specifically at 2-thiouridine, were found in certain bacterial species but not in others, and eukaryotic RNA and the presence of the gene sufY was correlated with geranylation across species [121]. The addition of this isoprenoid also has been attributed to SelU and often occurs in tRNA containing a carboxymethylaminomethyl (cmnmn) at 5-uridine, suggesting a coordination of these 5- and 2-uridine modifications through multienzyme biosynthetic pathways [122]. Of particular interest is the rhodanese homology domain of SelU, given the utility, in such motifs, in sulfurtransferase catalysis [122,123]. These conserved residues may provide insights into the rational design of an antibiotic.

In addition, aminoacyl tRNA synthetases have been proposed as potential drug targets for malaria and tuberculosis [124]. These enzymes catalyze the aminoacylation of tRNAs with cognate amino acids [125]. Aspartyl-tRNA synthetase has been explored as a potential drug target because of its structural differences from that of humans [126].

Several of the 36 Plasmodium falciparum aminoacyl-tRNA synthetases have been investigated as drug targets, including prolyl-tRNA synthetase (PRS) [126]. Jain et al. (2014) obtained a crystal structure of this parasite’s prolyl-tRNA synthetase and found some contrasts with the human glutamyl-prolyl-tRNA synthetase [127]. The former’s structure in 3 Å resolution revealed an open state upon ATP-binding and an asymmetric dimer interface, owing to a flexible segment extending into these ATP-binding pockets [127]. Structural analyses have revealed key differences between parasite and human enzymes that can be exploited for drug design.

## 3. Part III: RNA as a Therapeutic

The biological versatility of RNA, including its interactions with nucleic acids and proteins, makes it suitable as a novel class of therapeutics. Several RNA-based treatments have been approved, including anti-sense oligonucleotides (ASOs), RNA interference (RNAi), and CRISPR/Cas gene therapies [128,129]. Other approaches under investigation are RNA aptamers and the rational design of small molecule mimetics [130,131].

RNA folding patterns impart further functionality enzymatically and during translation.

Single-stranded RNA has been folded into varying conformations, performing biological tasks [132]. RNA can form distinct secondary and tertiary structures through complex intramolecular base-pairing and complementary interactions between different strands [133].

### 3.1. ASOs and siRNAs Silence Genes

Antisense oligonucleotides are short synthetic strands of DNA oligonucleotides designed to bind RNA and induce gene silencing [134,135]. Both in vitro and in vivo studies have demonstrated the efficacy of this approach [136,137]. ASOs bind to RNA in an antisense fashion, forming an RNA/DNA heteroduplex. The resulting double-stranded region of RNA is now a substrate to RNase H–an enzyme that removes RNA strands [138]. ASOs are also effective at manipulating alternative splicing through either exon skipping or exon inclusions [139]. The former can be useful for correcting frameshift mutations [140], whereby an ASO binds its target pre-mRNA and corrects the mutation, producing a short functional protein [141]. Conversely, exon exclusion methods involve ASOs attaching to the pre-mRNA to prevent spliceosomes and splicing factors from binding [142].

Despite the promise of gene therapies targeting disease at their source rather than upstream pathways [136,137], greater headway needs to be made prior to ASOs becoming a mainstay treatment plan.

RNA interference (RNAi) is another approach to silence disease-related genes, employing siRNAs [143]. The process involves introducing double-stranded siRNAs into cells, where they are incorporated into the RNA-induced silencing protein complex (RISC) [144]. Next, the strands are separated, and the guide strand ushers the RISC to its mRNA target for enzymatic digestion [145]. This technique can be applied to genetic disorders such as hypercholesterolemia, in which the drug Inclisiran targets PCSK9 mRNA to lower LDL cholesterol levels through the recycling of LDL receptors [146,147].

Considerations in the delivery of such RNA therapeutics include maintaining its structural integrity while routing its intracellular target. The chemical modifications of ASOs, such as the thiolation of the phosphate backbone at non-binding sites, adding 2′O-methyl/methoxyethyl groups [148], and employing gapmers, which also imparts stability by using DNA as central segments, facilitate cellular uptake [149].

Two of the three FDA-approved siRNA therapeutics are N-Acetylgalactosamine (GalNac)-based, meaning amino-sugar derivatives are conjugated to these siRNAs, designating them for the liver [150]. There, Givosiran alleviates acute hepatic porphyria and Inclisiran reduces cholesterol levels [151]. The other FDA-approved therapeutic of this class, Patisiran, uses a lipid nanoparticle (LNP) delivery method to treat hereditary transthyretin amyloidosis [152].

### 3.2. CRISPR/Cas9 and Gene Editing

CRISPR/Cas is a popular genome editing tool that can alter RNA as well as DNA [153]. RNA is the preferrable target because it bypasses the ethical concerns of permanently altering the genetic code [154]. Zhang et al. (2019) developed the RNA base-pair editing systems, REPAIR, which enables A to I(G) replacement, and RESCUE, which replaces C with U [154,155].

Therapeutic attempts at attenuating toxic RNA in DM1 mouse models include the CRISPR/Cas9-mediated genome editing of the expanded CTG repeats [156]. Another approach inserts polyadenylation signals upstream of the CTG repeats to reduce the expression of CUG-repeat-containing transcripts [157].

CRISPR/Cas can also be useful in fighting viral infections. For example, PAC-MAN is a CRISPR/Cas13 system has shown promise in targeting and destroying RNA in influenza and coronaviruses [158]. Similarly, CasRx is also an artificial enzyme that can target RNA; however, it can make specific cuts to RNA instead of outrightly destroying RNA [159,160].

The modes of delivering this gene editing complex include viral vectors [161], exosomes [162], various nanoparticles, and lipid nanoparticles (LNPs) [163], in which the Cas mRNA and gRNA are encapsulated to facilitate delivery [164]. For example, Pfizer’s COVID-19 vaccine is an mRNA administered within an ionizable lipid known as ALC-0315 [165].

### 3.3. Small Molecules as Inhibitors

Small molecules are readily finetuned through derivatization with various functional groups, and their small size leads them to targeting biomolecules implicated in RNA-based diseases [166,167]. RNA itself has multiple levels of structural organization, thereby affording a high specificity to a properly tailored small molecule [168]. Consequently, small molecules can disrupt RNA–protein interactions to prevent metastasis [169] or inhibit bacterial translation to stop infection, as referenced earlier with regards to SelU. Examples of the former include targeting splicing factors, such as SF3B1, PRPF8, and U2AF1, which could potentially correct aberrant splicing in cancer cells, thereby preventing further oncological effects [170,171,172].

Currently, the drugs Risdiplam, Branaplam, and Indisulam, among others, are under consideration for their potential in cancer treatments [173,174]. Indisulam is especially promising due to its ability to kill cancer cells directly by degrading the splicing factor, RBM39 [174].

The convenience of the oral administration and shorter half-lives of small molecules, as well as their longer shelf-life, lower manufacturing costs, and conduciveness to traditional high-throughput screening, highlights their therapeutic potential [175].

Aiding in the delivery of small molecules into cells or specific tissue-types are polymeric nanoparticles, lipid-based systems [176], and GalNAc, which are tailored to the asialoglycoprotein receptors (ASGPR) of liver hepatocytes [177,178].

### 3.4. RNA Aptamers Offer Variability

While the majority of this review has, thus far, focused on RNA therapeutics as targeting genes or other nucleic acid targets in an antisense fashion, these are not the limits of their potential. Most notably, RNA aptamers are a promising therapeutic that can bind not only to nucleic acids, but also to organic compounds, proteins, and even whole cells [130,179]. They can target a variety of molecules with high affinity and specificity in a similar fashion to antibodies. Furthermore, by modifying the backbone or nucleic acids, one can manipulate an aptamer’s half-life, affinity, and even its function [180,181].

**Figure 5:**
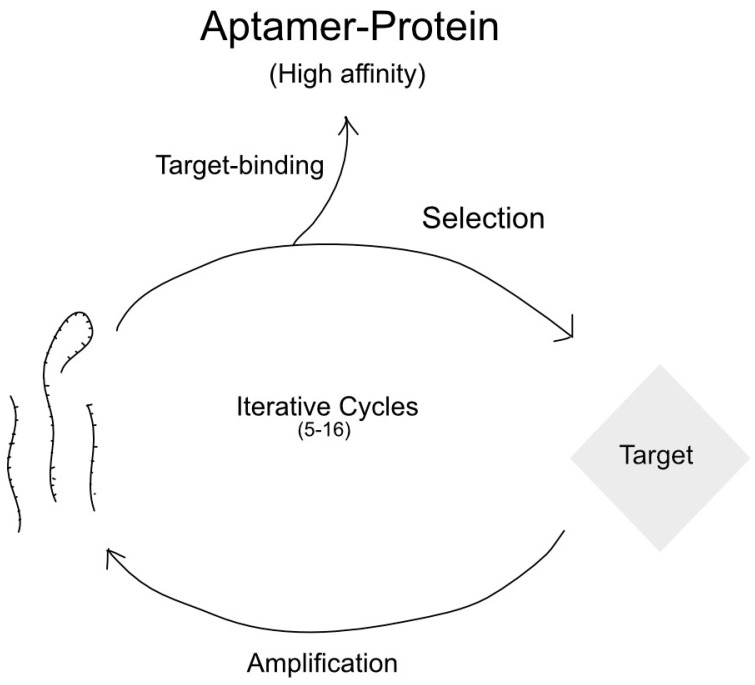
Schematic of SELEX procedure.

RNA aptamers work by intramolecularly hybridizing into a unique 3-dimensional structure, depending on the nucleic acid sequence [182]. Once formed, they can bind to their target and induce a variety of effects, such as inhibition or marking for destruction [183]. Due to their immense variability in structure, aptamers for a particular target must be selected for through the systematic evolution of ligands by exponential enrichment (SELEX) [184]. While there are many different types, the basic idea is that a large pool of RNA oligonucleotides are allowed to fold into a defined 3-dimensional structure, after which they are exposed to the target of interest [185,186]. The subset of oligonucleotides that bind is purified and amplified as potential aptamers [186]. This process is illustrated schematically in Figure 5. Because the 3-dimensional structures of aptamers vary with nucleic acid sequences, it is theoretically possible to design an aptamer against any molecular target [130,187,188].

Currently, there is only one FDA approved aptamer, under the name Pegnatib, to treat neovascularization, which is responsible for 90% of vision loss in age-related macular degeneration (AMD) [189,190]. Neovascularization is caused by the production of too much of the vascular endothelial growth factor (VEGF), which increases the proliferation of new blood vessels in the choroid, leading to vision loss [191]. Pregnatib acts as a potent inhibitor to VEGF165, the isoform primarily responsible for neovascularization, and has been shown to slow, and even prevent, further visual damage [192]. Other aptamers that act to inhibit alternate isoforms of VEGF responsible for more types of vision loss, such as that in diabetes, are being developed [193,194]. Many others could act as potential viral or cancer therapeutics. Pegnatib is the only RNA aptamer approved by the FDA because many of the same challenges of other RNA therapeutics, such as degradation by nucleases and cell permeability, apply to aptamers [195]. However, aptamers show incredible therapeutic potential [196].

The delivery is consistent with other oligonucleotide therapies, with the advantage of using SELEX for optimization of modifications, of which 2′-O methyl, 2′-fluoro, and phosphorothioate-linkage modifications proved efficacious [197,198]. Moreover, the protection from nucleases is afforded by enclosing the modified oligonucleotide within liposomes or LNPs [199]. Alternate methods of delivery include viral vectors [200], polymeric carriers [201], and metallic nanoparticles [202].

## 4. Conclusions

The evolution of RNA from its role as life’s original catalyst and genetic material into a behemoth of RNA-types and protein-coding processes has opened an avenue to its therapeutic development. This may entail targeting certain enzymes involved in splicing, RNA-folding, or post-translational modifications, or employing RNA itself as a therapeutic to modulate gene expression. RNA aptamers may even act as chemical antibodies. The flexibility of this molecule offers advantages over the traditional small molecule approach to pharmaceuticals, including their nucleotide-mediated selectivity and variety of applications by modification. This has implications in modulating gene expression and the treatment of a wide variety diseases.

Investigations into treating these disorders have illustrated the breadth of RNA-based therapeutic approaches at our disposal once the relevant biomarkers and mechanisms are understood. These include gene editing, the polyadenylation-mediated reduction in the expression of CUG-repeat-containing transcripts, the degradation of CUG-repeat RNA using antisense oligonucleotides or siRNAs, the targeting of small molecule inhibitors to the MBNL–CUG RNA interaction, and combinatorial antibody therapy.

The same repertoire of techniques can be applied to other pathologies, especially those pertaining to ribosome biosynthesis and its role in translation. The former is ramped up during cancer, rendering its mechanism of production an appealing means to deny cancerous cells a necessary commodity. Likewise, viruses commandeer the latter by imparting post-translational modifications on host-proteins, making the plethora of ribosomal and associated proteins plausible targets. Further, the pattern of RNA-folds and tRNA-modifications necessary to their functions can also provide insights into a therapeutic’s rational design.

## Data Availability

Not applicable.
